# Maternal Vitamin D Deficiency in Mice Increases White Adipose Tissue Inflammation in Offspring

**DOI:** 10.3390/cells11132024

**Published:** 2022-06-25

**Authors:** Nicole Haroun, Imene Bennour, Eva Seipelt, Julien Astier, Charlene Couturier, Lourdes Mounien, Jean-François Landrier

**Affiliations:** C2VN, UMR 1260 INRAE/1263 INSERM, Aix Marseille Université, 27 Bd Jean Moulin, CEDEX 05, 13385 Marseille, France; nicole.haroun@univ-amu.fr (N.H.); imene.bennour@univ-amu.fr (I.B.); seipelt.eva@gmail.com (E.S.); julien.astier@univ-amu.fr (J.A.); charlene.couturier@univ-amu.fr (C.C.); lourdes.mounien@univ-amu.fr (L.M.)

**Keywords:** vitamin D, maternal vitamin D deficiency, offspring, white adipose tissue, inflammation, NF-kB, p38

## Abstract

Vitamin D is acknowledged to play an important biological and metabolic role in adipose tissue, which is also the main storage site for this vitamin. Its anti-inflammatory effect in adipocytes and adipose tissue has notably been highlighted in adult mice. This vitamin is also crucial during fetal development since maternal vitamin D deficiency is suspected to program future metabolic disorders. Based on these observations, the aim of this study was to evaluate the consequences of maternal vitamin D deficiency (VDD) on white adipose tissue inflammation in adult offspring fed with normal or obesogenic diet (high-fat diet). White adipose tissue morphology, RNA and miRNA expression profiles, and signaling pathways were studied in adult males and females. In males, a HF diet coupled with maternal VDD increased expression of RNA and miRNA linked to inflammation leading to over-representation of inflammatory pathways. Interestingly, genomic and epigenetic profiles were associated with activation of the NF-kB signaling pathway and adiposity index. In females, no major modulation of inflammatory pathways was observed under VDD, contrarily to males. We concluded that maternal VDD coupled with HF diet activated inflammatory pathway in adipose tissue of the offspring, in a sex-dependent manner. Such activation is strongly related to activation of NF-kB signaling and increased adiposity only in males.

## 1. Introduction

Vitamin D (VD) deficiency (VDD), defined by 25-hydroxy vitamin D (25(OH)D) plasma levels below 50 nmol/L has become a worldwide public health problem that affects in particular women of childbearing age, pregnant women, or those breastfeeding [1]. This vitamin, which can be obtained from food or produced endogenously [2], is thought to play an essential function in fetal development but also throughout the life. In pregnant women, VDD is associated with an increased risk of pre-eclampsia [3]. The VDD is associated with a delay of intrauterine growth or SGA (small gestational age) [4], a decrease in head circumference [5] and body size, and low birth weight in the newborn [6]. In 4–6-year-old children born of VDD women, an increase in body fat [7,8], BMI (body Mass Index) and waist circumference has been observed [9]. In children aged 6 and 9.5 years, lean body mass percentages decrease [5], whereas insulin resistance increases [10]. In agreement, we recently showed in mice that maternal VDD induced low body mass in juvenile males, which was related with an increase in energy expenditure, but not in females [11]. In adults submitted to a normal diet, maternal VDD did not massively affect body mass, adiposity index, or insulin resistance in both males and females. Nevertheless, under high fat diet, we observed a rise in adiposity and insulin resistance in males, but not in females. Such observation strongly, together with others [12,13,14,15], supports an impact of VDD on adipose tissue physiology in perinatal context [16].

Of note, the relationship between vitamin D and adipose tissue physiology has been already demonstrated in adulthood, where VD anti-inflammatory effects in adipocyte and adipose tissue have been demonstrated in the context of obesity [16,17]. Indeed, obesity is classically associated with a low-grade inflammatory status in adipose tissue, resulting notably from the activation of several inflammatory signaling pathways including NF-B and p38/mapk [18]. The activation of these pathways contributes to the production of pro-inflammatory cytokines, chemokines, and miRNA synthesis in adipocytes but also in stroma vascular fraction, contributing to leucocyte infiltration in the adipose tissue [18,19,20]. Interestingly, we and others have demonstrated an anti-inflammatory effect of vitamin D on adipocytes, involving an inhibition of NF-B signaling pathway and p38 protein phosphorylation and leading to a significant decrease in cytokines, chemokines, and miRNA expression and release [21,22,23,24,25,26].

Altogether these data show a strong inhibitory effect of vitamin D and adipose tissue inflammation in adults, no data are presently available about the programming effect of maternal VDD on adipose tissue inflammation of the offspring. Therefore, the aim of the present study was to determine the impact of maternal VDD in mice (25(OH)D) plasma levels below 10 ng/mL) combined or not with an obesogenic environment on the sex-specific response of inflammation of white adipose tissue in adult offspring.

## 2. Material and Methods

### 2.1. Animal Experiments

The protocol was approved from an ethical point of view by the Aix-Marseille University Ethics Committee and the French Ministry of Research (APAFIS#1300-2015072112279135). Thirty female and ten male C57BL/6JRJ mice were obtained from Janvier Labs (Le Genest-Saint-Isle, France), fed ad libitum during the 1-week acclimation period with control food (chow diet A04 from Safe-diets, Augy, France) and given full access to drinking water. The animals were kept at 22 °C with a 12 h light/12 h dark cycle and a humidity level of 20%. Female mice (15 per group) were mated with males (5 per female group) after being randomly allocated to one of two experimental groups based on the diet: control (AIN-93G with vitamin D3, 1.0 IU/g) or vitamin D-depleted (AIN-93G without vitamin D3, 0.0 IU/g) for 8 weeks, as previously described [11]. After delivery, all females were fed control diet (AIN-93G) until the offspring were weaned. The females’ litter size was reduced to six pups. To avoid mother cannibalism and perinatal stress, the offspring’s body weight was measured weekly from the time they were weaned until the end of the trial. Males and females of the offspring were randomly assigned to receive either a Low-Fat diet (AIN-93M Maintenance Purified Diet) or a High-Fat diet (DIO Rodent Purified Diet w/45 percent energy from fat) for 8 weeks at 6 weeks of age. The impact of maternal diet (CTRL vs. VDD) and adult diet on offspring mice (males and females) was investigated using eight groups of mice (males and females) (LF vs. HF).

Overnight fasted mice were euthanised by cervical dislocation, and tissue samples were taken, weighed, and kept at −80 °C.

### 2.2. RNA Extraction Real Time PCR and RNA Sequencing

TRIzol reagent (Thermo Fischer Scientific, Les Ulis, France) was used to extract total RNA from retroperitoneal adipose tissue as previously described [27,28]. Total RNA from three mice per group were used to prepare an RNA-seq library using the Illumina TruSeq Stranded mRNA kit. On the Illumina NextSeq 500 sequencer, libraries were sequenced paired-end. Sickle was used to eliminate reads having a phred score of less than 20 and a length of less than 25 bp (v1.33). MultiQC was used to assess the quality of the trim reads (v1.0). Trim readings were aligned with the STAR aligner (v2.7.0d) with the “outFilterMismatchNoverLmax” and “outFilter-MultimapNmax” options set to 0.08 and 1, respectively. Cufflinks (v2.2.1) was used to find transcripts, with the “library-type” option set to fr-firstrand and a GTF file obtained from GENCODE (“Comprehensive gene annotation”, vM1) serving as the genomic annotation. Cuffmerge was used to integrate the GTF files created by Cufflinks for each sample.

Unknown transcripts (class code “u”) were identified using the “class code” supplied by Cuffmerge to each transcript. For further analysis, only de novo transcripts with counts greater than 0 in at least one RNA-seq sample were preserved. These newly created transcripts were joined with the GENCODE GTF file to create the final genomic annotation, which was then quantified using FeatureCounts (v1.6.1). DESEQ2 was used to compare gene expression patterns between conditions. Reads from Watson and Crick strands were picked using SAMtools (v1.9) and sent into the RseQC program suite’s bam2wig.py script to build bigwig files (v2.6.4). The IGV genome browser was used to visualise RNA-seq profiles. RNA-seq data are available at GEO (accession number: GSE206372).

### 2.3. Gene Ontology (GO) and Ingenuity Pathway Analysis (IPA)

Differential expression data were filtered using the following parameters: *padj* < 0.01 (*p*-value corrected for multiple tests using Benjamini and Hochberg method) and fold change (FC) FC > 1.5 or <−0.66. Those gene lists were implemented in Gene Ontology (GO) and Ingenuity Pathway Analysis (IPA) software to highlight metabolic pathways differentially impacted by the maternal diet and subsequent LF/HF diet. Pathways were manually curated to retain only those related to inflammation in our study. Finally, quantitative analyses were performed to characterise the influence of the maternal diet and/or the adult diet in inflammation pathways in the offspring.

### 2.4. Evaluation of miRNA Expression

The miScript PCR array (Qiagen, Coutraboeuf, France) was used for miRNA expression in white adipose tissue as previously described [25]. Reactions were performed in a volume of 12.5 µL containing 6.5 µL of 2× QuantiTect SYBR Green PCR Master Mix (Qiagen, Coutraboeuf, France), 1.25 µL of 10× miScript Universal Primer (Qiagen, Coutraboeuf, France), in the presence of 250 ng of total RNA. After an initial incubation step of 15 min at 95 °C, the amplification reaction was performed over 40 cycles, comprising 3 steps (95 °C, 15 s; 55 °C, 30 s and 70 °C, 30 s). For each condition, expression was quantified from 5 biological replicates; replicates and SNORD68, RNU6-6P were used as endogenous controls in the comparative threshold cycle (Ct) method [29].

### 2.5. miRNA Target Prediction and Pathway Analysis

Inflammatory miRNA was selected and predicted mRNA target list was established using Targetscan (gene were selected with an Aggregate PC ≥ 90%) and mirDB (gene were selected with a target score ≥80%). The common gene list was subjected to computational analysis with IPA to identify biological processes associated with the miRNA inflammation-related pathways in the white adipose tissue. Quantitative analyses were conducted to characterise the influence of the maternal diet and/or the adult diet in inflammation pathways in the offspring.

### 2.6. NFκB Activation and p38/MAPK Activation

Retroperitoneal adipose tissue homogenates were prepared as previously described [30]. The levels of phosphorylation of NFkB p65 (Phospho) (pS536) and NFkB p65 (Total) were measured using the ELISA Instant One kit (eBiosciences SAS, Paris, France) according to the manufacturer’s instructions. NFkB p65 (Phospho/Total) werwase used to compare pathway activation in the various groups. The levels of phosphorylation of p38 MAPK (Phospho) [pT180/pY182 and p38 MAPK (Total) were measured using the ELISA Instant One kit (eBiosciences SAS, Paris, France) according to the manufacturer’s instructions. P38 (Phospho/Total) was used to compare pathway activation in the various groups.

### 2.7. Statistical Analysis

The data are presented as mean ± SEM. GraphPad Prism (version 9.3.1, GraphPad software LLC, San Diego, CA, USA)) was used to assess significant differences using an ANOVA followed by the Fisher’s LSD post hoc test. A statistically significant value of *p* < 0.05 was used.

## 3. Results

### 3.1. HF Diet and Maternal VDD Impact Body Weight and Adiposity Index

**Figure 1:**
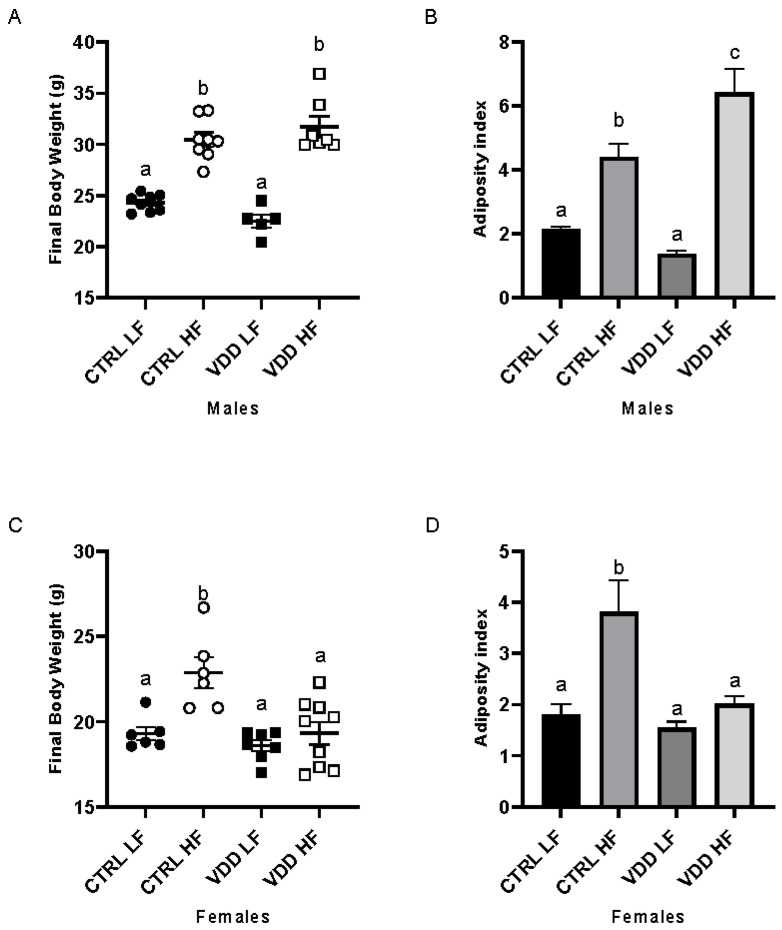
Body weight and adiposity index in males and females offspring. Body weight measured at the protocol end for the males and females offspring (**A**,**C**). Adiposity index of the offspring has been established for males and females (**B**,**D**). Values are presented as mean ± SEM. Bars not sharing the same letter were significantly different in Fisher’s LSD post hoc test. *p* < 0.05.

After euthanasia, CTRL LF and VDD LF males showed similar body weight; similarly, CTRL HF and VDD HF diet had equivalent body weight. A significant difference between the LF (both CRTL and VDD) and HF groups (both CTRL and VDD) (Figure 1A) was observed, with higher body weight in HF-fed groups (Figure 1A). For females, only CTRL HF had higher body weight (Figure 1C) compared with other groups (CTRL LF, VDD LF and VDD HF). Concerning the adiposity index, VDD HF males presented the highest adiposity index (Figure 1B) compared with the three other groups (CTRL LF, CTRL HF, and VDD LF males). The females CTRL HF showed highest adiposity index (Figure 1D) compared with other groups (CTRL LF, VDD LF, VDD HF females).

### 3.2. HF Diet and Maternal VDD Regulate Inflammation Pathways

In order to highlight the inflammatory response in the WAT, RNA-seq analyses were set up in order to study the expression profile of the genes in the WAT. Using the significant regulated genes (Table 1), two sets of data were employed. The first set looked at the effect of maternal VDD in the same diet conditions (i.e., CTRL-LF vs. VDD-LF and CTRL-HF vs. VDD-HF). The second data set evaluated the effects of a HF diet on the same type of maternal diet (CTRL-LF vs. CTRL-HF and VDD-LF vs. VDD-HF). 

**Figure 2:**
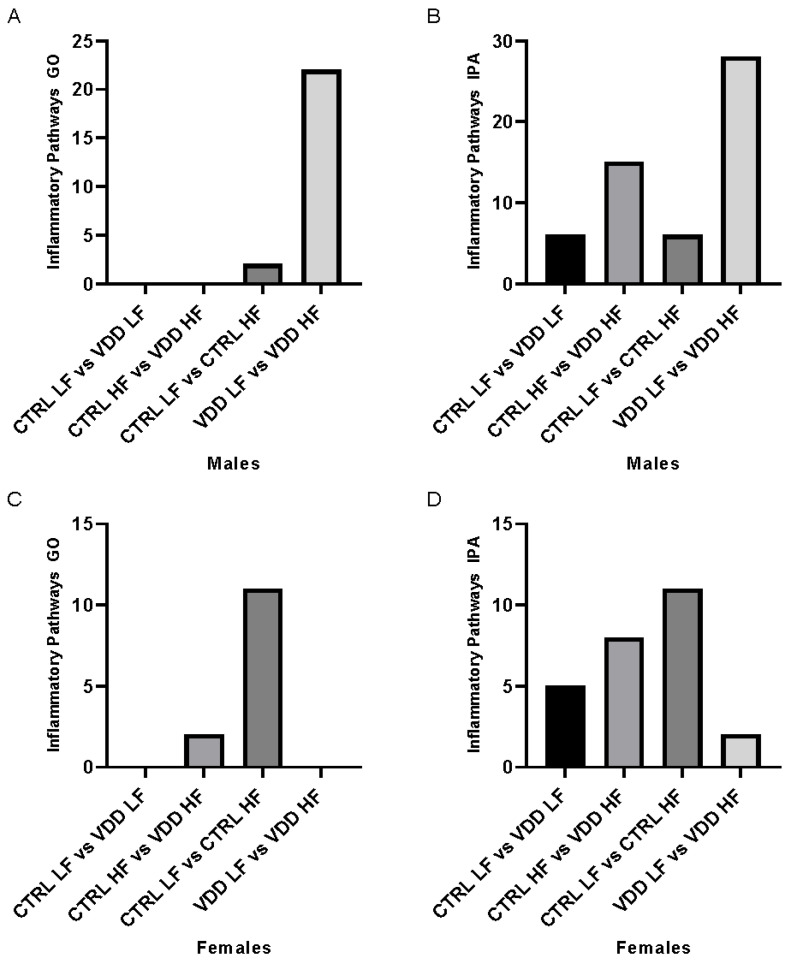
Inflammatory pathways linked to RNA profile in adipose tissue of the offspring. Quantitative representation of inflammatory pathways resulting in differential gene expression in retroperitoneal adipose tissue of the male offspring (**A**,**B**) and female offspring (**C**,**D**) using Gene Ontology (GO) and Ingenuity Pathway Analysis (IPA). Two sets of data were employed. The first is CTRL LF vs. VDD LF and CTRL HF vs. VDD HF. The second data is CTRL LF vs. CTRL HF and VDD LF vs. VDD HF. The genes were originally chosen based on the following criteria: *padj* < 0.01 and fold change FC > 1.5 or −0.66.

In a first set of analysis, we investigated the impact of maternal VDD. No pathway related to inflammation in both conditions, LF and HF diet (CTRL LF vs. VDD LF males and in CTRL HF vs. VDD HF males) were highlighted using GO analysis in males (Figure 2A). Using IPA, we selected inflammatory canonical pathways with determinant z-score. We highlighted 6 differentially expressed canonical pathways between CTRL LF and VDD LF males (Supplementary Table S1), and 15 differentially expressed canonical pathways between CTRL HF and VDD HF males (Supplementary Table S2). Three common pathways were found (Figure 2B). In females, in both conditions CTRL LF vs. VDD LF and in CTRL HF vs. VDD HF, using GO analysis, no pathway that triggers the inflammation in the LF situation and two inflammatory pathways in the HF diet were identified (Figure 2C; Supplementary Table S3). Using IPA, 5 differentially expressed canonical pathways between CTRL LF and VDD LF females (Supplementary Table S4), and 8 differentially expressed canonical pathways between CTRL HF and VDD HF females were identified (Figure 2D, Supplementary Table S5). Five pathways were found in common between the CTRL LF vs. CTRL HF and VDD LF vs. VDD HF.

Then, we analysed the impact of a HF diet on the same maternal vitamin D status background (CTRL or VDD). In males, 2 pathways related to inflammation were observed in CTRL LF vs. CTRL HF (Supplementary Table S6) and 22 pathways in VDD LF vs. VDD HF (Figure 2A, Supplementary Table S7). In these 2 analyses, the “response to cytokines” pathway was induced, but the upregulation in VDD HF vs. VDD LF condition was higher compared with the CTRL HF vs. CTRL LF condition as revealed by the number of genes involved and the associated *p*-value (120 genes vs. 67 genes, and 6.95 × 10^−8^ *p*-value vs. 9.48 × 10^−4^ *p*-value, respectively). The same analyses were performed using IPA software and similar results were obtained. Namely, when comparing CTRL LF vs. CTRL HF, 6 differentially expressed canonical pathways were highlighted (Supplementary Table S8) and 28 differentially expressed canonical pathways between VDD LF and VDD HF males (Supplementary Table S9). Four pathways were found in common between the CTRL LF vs. CTRL HF and VDD LF vs. VDD HF (Figure 2B). In females, GO analysis revealed 11 pathways related to inflammation in CTRL LF vs. CTRL HF (Supplementary Table S10) and none in VDD LF vs. VDD HF females (Figure 2C). Using IPA, 11 differentially expressed canonical pathways between CTRL LF and CTRL HF females (Supplementary Table S11) and 2 canonical pathways between VDD LF and VDD HF females were observed (Supplementary Table S12). No common pathway was found between the two groups (Figure 2D).

These gene lists were used in Gene Ontology (GO) and Ingenuity Pathway Analysis (IPA) tools to identify metabolic pathways that were affected differently by the maternal diet and the subsequent LF/HF diet. Pathways were manually filtered to keep those connected to inflammation. Finally, quantitative analyses were performed to determine the impact of the maternal and/or adult diets on inflammation pathways in offspring.

### 3.3. HF Diet and Maternal VDD Regulate miRNA Expression and Related Inflammatory Pathways

**Figure 3:**
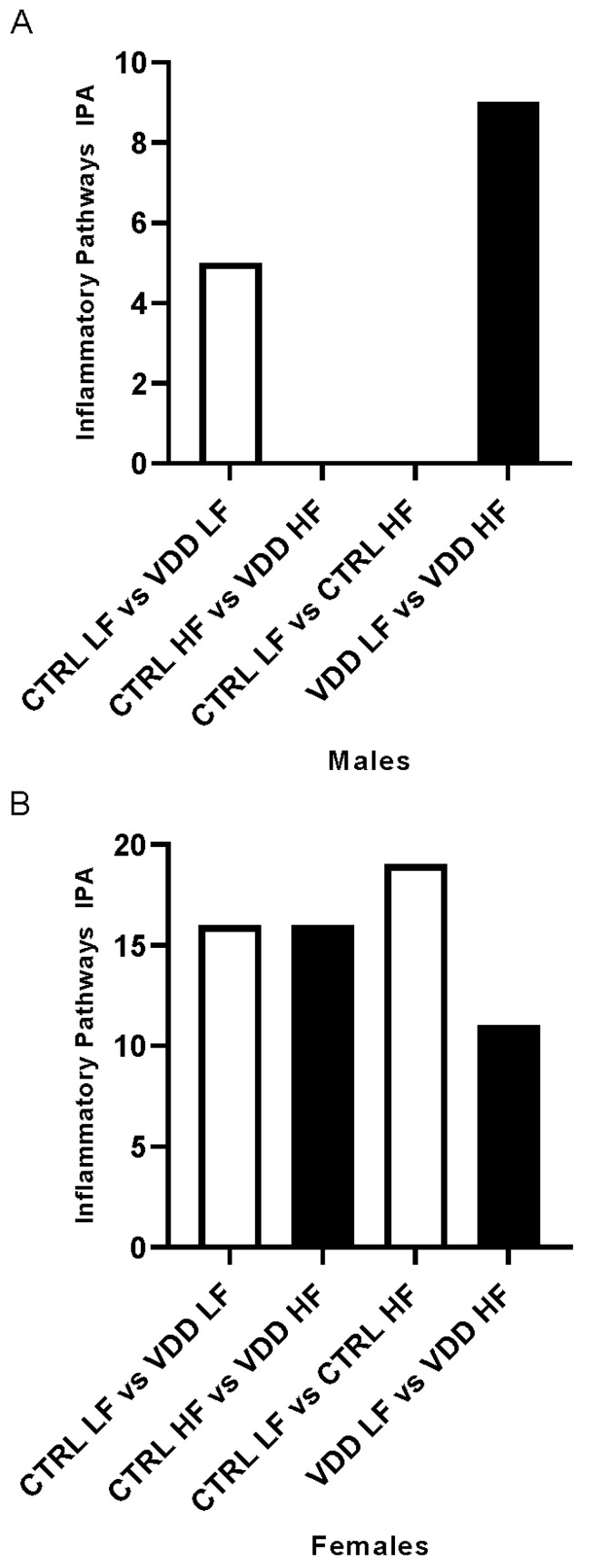
Inflammatory pathway linked to miRNA profile in adipose tissue of the offspring. Quantitative representation of miRNA expression in inflammatory pathways resulting in differential miRNA expression in retroperitoneal adipose tissue of the male offspring (**A**) and female offspring (**B**), using Ingenuity Pathway Analysis (IPA). Two sets of data were employed. The first is CTRL LF vs. VDD LF and CTRL HF vs. VDD HF. The second data set is CTRL LF vs. CTRL HF and VDD LF vs. VDD HF. Bars in white correspond to up-regulated pathways and bars in black for down-regulated pathways. TargetScan (genes were selected with an Aggregate PC 90%) and mirDB (genes were selected with a target score 80%) were used to identify inflammatory miRNA and create a predicted mRNA target list. The miRNA inflammation-related pathways in white adipose tissue were analysed using IPA. To assess the influence of the maternal diet and/or the adult diet on inflammation pathways in the offspring, quantitative analyses were carried out.

To go further in the identification of inflammation process, we investigated the effect of maternal VDD and/or HF diet on inflammation-related miRNA expression (Table 2, Supplementary Table S13) and related inflammatory pathways using IPA. The effect of maternal VDD was analysed in LF and HF conditions. In males, 5 pathways were obtained between CTRL LF and VDD LF (Supplementary Table S14) and no pathways were found in CTRL HF vs. VDD HF (Figure 3A). In females, the comparison CTRL LF vs. VDD LF females identified 16 inflammatory pathways (Supplementary Table S15) and 16 inflammatory pathways in CTRL HF vs. VDD HF (Figure 3B, Supplementary Table S16). Seven of these pathways were discovered to be common. The same methodology was implemented to investigate the effects of HF diet on the same type of maternal regimen. In IPA, the gene list revealed no pathways in CTRL LF vs. CTRL HF and 7 in VDD LF vs. VDD HF in males (Figure 3A, Supplementary Table S17). In females, 19 inflammatory pathway responses in CTRL LF vs. CTRL HF (Supplementary Table S18) and 10 inflammatory pathways responses in VDD LF vs. VDD HF were observed (Figure 3B, Supplementary Table S19), among which 6 were common.

### 3.4. HF Diet and Maternal VDD Activate NF-kB and p38

**Figure 4:**
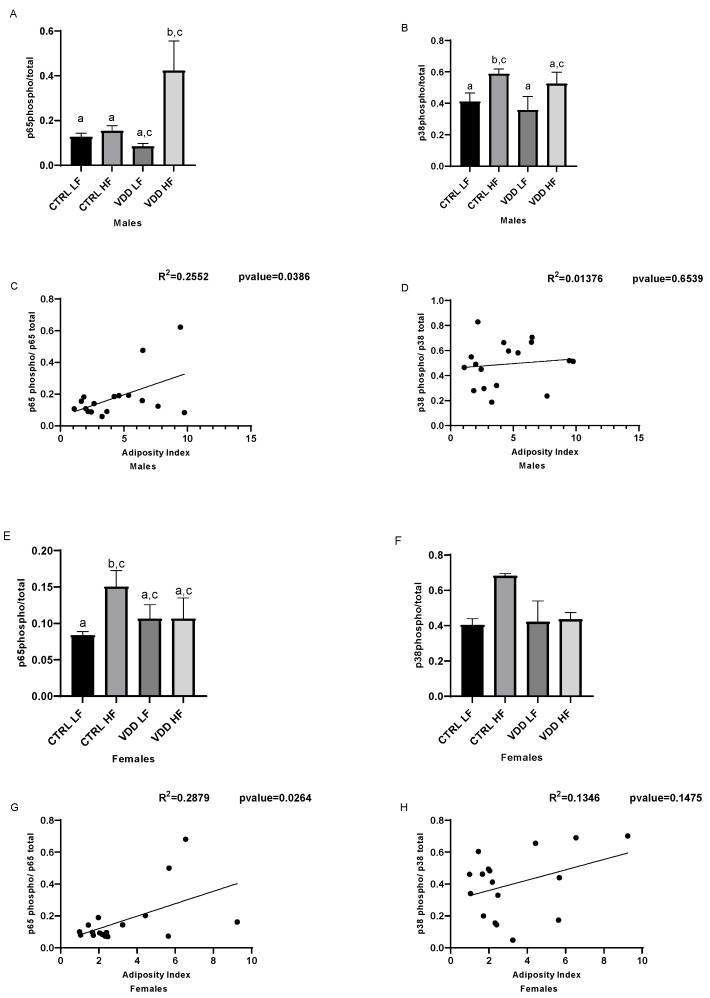
Phosphorylation levels of the NF-kB (p65) and p38 in adipose tissue of the offspring. Phosphorylation levels of p65 and p38 were evaluated using ELISA in males (**A**,**B**) and females (**E**,**F**). The data are expressed as relative expression ratios (p65phosphorylated/p65total and p38 phosphorylated/p38total). Values are presented as mean ± SEM. Bars not sharing the same letter were significantly different in Fisher’s LSD post hoc test. *p* < 0.05. Scatter plot of the correlation between adiposity p65 in males (**C**) and females (**G**), and scatter plot of the correlation between adiposity and p38 in males (**D**) and females (**H**), using Pearson r correlation, *p*  <  0.05 indicated that positive correlation was significant.

To unveil the molecules at the origin of inflammatory response in white adipose tissue, NF-kB and p38 signaling were investigated. Thus, p65 phosphorylation and p38 phosphorylation were quantified. Highest p65 phosphorylation was found in VDD HF males compared with other groups (Figure 4A) whereas p38 phosphorylation was significantly induced in CTRL HF males compared with other groups (Figure 4B). p65 phosphorylation in females was induced only in CTRL HF compared with other groups (Figure 4E), whereas p38 tended to be highly phosphorylated in CTRL HF but did not reach statistical significance (Figure 4F).

In both males (Figure 4C) and females (Figure 4G), a significant correlation was calculated between adiposity index and p65 phosphorylation, whereas no significant correlation was observed between p38 phosphorylation and adiposity index (Figure 4D,H).

## 4. Discussion

Maternal diet is now recognised as an important factor in fetal development and offspring metabolic programming [31], in line with the DOHaD concept [32]. This concept states that early life environment may impact the risk of developing chronic diseases, formerly referred to as non-communicable diseases, from childhood to adulthood. In other words, the DOHaD corresponds to fetal or developmental programming. The fact that vitamin D shapes adipose tissue inflammatory profile in adults prompted us to test its ability to program adipose tissue inflammation in offspring.

C57BL/6J mouse strain has been found to be a *bona fide* interesting model for obesity and associated dysfunctions, including metabolic inflammation. To study the effect of maternal VDD, female mice were fed with a vitamin D-free diet for at least 8 weeks premating and during the gestation period. After delivery, mice were all fed with control diet. At the age of 6 weeks, the offspring were randomised and received a low-fat (7% of total energy from fat) or high-fat diet (45% of total energy from fat) for 8 weeks to study the combined effect of the diet and maternal VDD on retroperitoneal adipose tissue inflammation.

To evaluate the combined effect of VDD and HF, a morphological analysis was established. In agreement with already published data of our team [11], we observed in this model an increase in the body weight under HF diet in both CTRL and VDD males. Adiposity index, which is used as a good marker of fat pads accumulation, independently of total body weight, was not affected by VDD in the LF condition, but was dramatically increased in the HF condition compared with the other group (CTRL LF, CTRL HF, and VDD LF). In females, a higher body weight and adiposity index was observed in CTRL HF compared with the other groups (CTRL LF, VDD LF and VDD HF). Surprisingly, the VDD HF group had a similar body weight and adiposity index to the LF groups (CTRL LF and VDD LF), thus demonstrating a strong sex-specific metabolic response.

Such increase in adiposity index in males and females groups prompted us to evaluate consequences in terms of inflammation, since it is well-established that adiposity or obesity is strongly associated with an increase of adipose tissue inflammation [18]. To study such inflammatory response, an RNA-seq approach was conducted on retroperitoneal adipose tissue and impacted metabolic pathways were estimated using Gene Ontology enrichment analysis (GO) and Ingenuity pathway analysis (IPA). After curation, only pathways related to inflammation were maintained in our quantitative analysis, based on the number of pathways impacted.

When comparing the impact of maternal VDD, under LF diet (CTRL LF vs. VDD LF) in males, only two linked inflammatory pathways were found to be significantly different, including a downregulation of “LPS/IL-1 Mediated Inhibition of RXR Function”, suggesting thus that under normal diet, the effect of VDD in males is very minor or null. However, under HF diet (CTRL HF vs. VDD HF) in males, we observed that two important pathways for adipose tissue biology [20], i.e., “NF-κB activation by viruses” and “p38 MAPK Signaling” were upregulated, suggesting that VDD exacerbated inflammation mediated by HF diet. To highlight the impact of a HF diet on inflammatory process in both CTRL and VDD situation, we compared the CTRL and the VDD in both LF and HF diets. An induction of the inflammatory response in CTRL LF vs. CRTL HF was observed, but this induction was even more remarkable in VDD males subjected to an HF compared to LF diet. Indeed, in these two conditions, the “response to cytokines” pathway was induced. This is a very important pathway of inflammation that includes 757 genes and recapitulates most on the inflammatory response. In CTRL LF vs. CTRL HF comparison, 67 genes differentially regulated while in VDD LF vs. VDD HF comparison, 120 genes were regulated, suggesting that the response is much more marked in animals born from VDD mice than in animals born from CTRL mice. This qualitative response was confirmed by a statistic *p*-value more important in the VDD animals than in the CTRL animals. Interestingly, such proinflammatory profile of VDD HF males group perfectly fit with adiposity index which is more pronounced in these animals. Altogether theses data suggest an additional effect of VDD and HF in males regarding the mediation of adipose tissue inflammation.

In females, in the response to maternal deficiency, the same response in both CTRL LF vs. VDD LF and CTRL HF vs. VDD HF groups was characterised by an upregulation of “NRF2-mediated Oxidative Stress Response”. In CTRL females, under HF diet (CTRL LF vs. CTRL HF) the “chemokine signaling” was upregulated as well as “response to cytokine”, in which 106 genes were upregulated in the CTRL HF females. It is noteworthy that that animals also displayed higher adiposity supporting the association between adiposity and inflammation. In agreement, in the VDD LF vs. VDD HF analysis, no inflammatory pathway was overexpressed and no discrepancy in terms of adiposity was observed between these groups.

MicroRNAs (miRNAs) are involved in adipose tissue inflammation during obesity [19,33,34,35]. This led us to investigate the miRNA profile and predicted associated pathways. In our study, for the first time, it was demonstrated that maternal VDD deregulated miRNA expression profiles in both male and female offspring. Moreover, this regulation also appeared to be sex-dependent, with a higher number of miRNAs modulated in females. The reason for such a discrepancy will require further investigations. To go further, predicted mRNA targets of the deregulated miRNAs were collected and enrichment analysis of pathway was conducted using IPA. In males, only one up-regulated miRNA was discovered in the CTRL LF vs. VDD LF, leading to the negative enrichment of five pathways, including “IL6 Signaling” and “ILK Signaling”, suggesting a decrease in inflammatory status in VDD LF males compared with CTRL LF males. Such an observation could be explained by the lower (even if not significant) adiposity index between VDD LF and CTRL LF males. In agreement, between VDD LF and VDD HF, five miRNAs were down-regulated, leading to positive enrichment of seven inflammatory-related pathways, including “p38 MAPK” and “Chemokine Signaling”, thus demonstrating at the miRNA level, the pro-inflammatory tone is also observable in male born from VDD mice and fed with HF diet. The comparison CTRL LF vs. VDD LF in females resulted in four up-regulated miRNA and subsequently 16 putative target pathways including “ERBB4 Signaling” and “p38 MAPK Signaling”, an important inflammatory pathway, as previously mentioned, supporting at the miRNA level that VDD is associated with a decrease of inflammatory status, even if no morphometric parameters, including adiposity support, are assumed. The origin of such a phenotype will require further investigations. Nevertheless, it is noteworthy that mR-146a-5p and mir-322-5p were both up-regulated within the four up-regulated miRNAs. These miRNAs are known to display anti-inflammatory effect: miR-146a-5p suppresses the inflammatory response in human adipocytes [36] and miR-322-5p targets NFkB1 and suppresses inflammatory cytokine production while promoting cell proliferation in LPS-stimulated murine macrophages [37], suggesting that even if pathway analysis revealed an upregulation of inflammatory pathways, miRNA at the origin of these putative enriched pathways display anti-inflammatory response. Similarly, in the CTRL HF vs. VDD HF in females, 16 pathways were predicted to be up-regulated on the basis of the down-regulation of one miRNA. In the VDD LF vs. VDD HF females, ten pathways were predicted to be up-regulated on the basis of the downregulation of three miRNA and in the CTRL LF vs. CTRL HF, 19 pathways were down-regulated (driven but the regulation of one miRNA). Surprisingly, all these data appeared to be inconsistent with morphometric parameters and notably with adiposity index. The origin of such inconsistence is presently unclear but could rely on the predictive approach implemented, which will require further optimisations.

Concerning molecular mechanisms, RNA seq and miRNA analysis as well as bibliography strongly converge to the putative role of NF-kB and p38/MAPK signaling pathways. Indeed, NF-kB and p38 are known to play a major role during adipose tissue inflammation [38,39]. Furthermore, we and others have demonstrated that the ability of VD to blunt inflammation in adipocytes and adipose tissue was linked to an inhibition of phosphorylation of these two signaling pathways [21,26,40,41,42]. To test the hypothesis of an NF-kB and/or p38 signaling involvement, we measured the phosphorylation level of p65 and p38 in adipose tissue. No activation of p65 or p38 in the VDD LF compared to CTRL LF males was observed, suggesting that the VDD alone does not activate inflammatory pathways in agreement with pathways analyses. Interestingly, a massive phosphorylation of p65 was observed, suggesting an additional effect of VDD and HF diet in male, as observed for inflammatory pathways and adiposity index. A significant induction of p38 phosphorylation was observed in the CTRL HF group, which is coherent since this pathway is known to be inducted during obesity [39]. Nevertheless, no impact of VDD was observed on the pathway. In females, p65 and p38 were not induced in VDD mice both under LF or HF diet, the only induction of p65 being found in CTRL HF that displayed the higher adiposity and activated inflammatory-related pathways.

The origin of the mechanisms that triggered the effect of vitamin D deficiency on inflammatory process in adipose tissue remains elusive. Nevertheless, we can speculate the epigenetics mechanisms could explain, at least in part, the observed phenotype. It is notably well-established that several Dups proteins are involved in dephosphorylation of stress-activated kinases [43]. It would be of interest to evaluate the epigenetic landscape of genes coding for these proteins. Clearly, further work is mandatory to elucidate the epigenetic mechanisms that may explain the observed regulations.

To summarise, our data established a link between the increased adiposity in males born from VDD mice and fed with a HF diet, which correlates with induction of mRNA and miRNA linked to inflammation and activation of p65 phosphorylation, whereas such a relationship was not observed in females. These data add to our understanding of maternal VDD influence on the offspring, particularly its predisposition to long-term metabolic health issues. It also highlights the sex-specific adipose tissue and inflammatory response that must be considered in terms of public health.

## Figures and Tables

**Table 1:** The total number of differentially expressed mRNA genes, including up-regulated and down-regulated genes, in both males and females, was determined using data filtering with *padj* < 0.01 and FC > 1.5 or <−0.66.

	Total mRNA Differentially Expressed	Up-Regulated	Down-Regulated
Males			
CTRL LF vs. VDD LF	348	146	20
CTL HF vs. VDD HF	907	650	257
CTRL LF vs. CTRL HF	1065	581	484
VDD LF vs. VDD HF	2406	1315	1091
Females			
CTRL LF vs. VDD LF	1323	820	503
CTL HF vs. VDD HF	1451	719	732
CTRL LF vs. CTRL HF	2234	1250	984
VDD LF vs. VDD HF	363	185	178

**Table 2:** The total number of differentially expressed miRNA that enters White adipose tissue inflammatory pathways, including up-regulated and down-regulated miRNA, in both males and females, was determined using data filtering with *p* < 0.05.

	Total miRNA Differentially Expressed	Up-Regulated	Down-Regulated
Males			
CTRL LF vs. VDD LF	1	1	0
CTL HF vs. VDD HF	0	0	0
CTRL LF vs. CTRL HF	0	0	0
VDD LF vs. VDD HF	5	0	5
Females			
CTRL LF vs. VDD LF	4	4	0
CTL HF vs. VDD HF	1	0	1
CTRL LF vs. CTRL HF	1	1	0
VDD LF vs. VDD HF	3	0	3

## Data Availability

RNA seq data are available on GEO (accession number: GSE206372).

## Supplementary Materials

The following supporting information can be downloaded at: https://www.mdpi.com/article/10.3390/cells11132024/s1, Supplemental Table S1: CRTL LF vs. VDD LF male, IPA; Supplemental Table S2: CTRL HF vs. VDD HF male, IPA; Supplemental Table S3: CTRL HF vs. VDD HF female, GO; Supplemental Table S4: CTRL LF vs. VDD LF female, IPA; Supplemental Table S5: CTRL HF vs. VDD HF female, IPA; Supplemental Table S6: CTRL LF vs. CTRL HF male, GO; Supplemental Table S7: VDD LF vs. VDD HF male, GO; Supplemental Table S8: CTRL HF vs. CTRL LF male, IPA; Supplemental Table S9: VDD LF and VDD HF male, IPA; Supplemental Table S10: CTRL LF vs. CTRL HF female, GO; Supplemental Table S11: CTRL LF and CTRL HF female, IPA; Supplemental Table S12: VDD LF and VDD HF female, IPA; Supplemental Table S13: miRNA differentially regulated; Supplemental Table S14: CTRL LF vs. VDD LF male, IPA; Supplemental Table S15: CTRL LF vs. VDD LF female, IPA; Supplemental Table S16: CTRL HF vs VDD HF female, IPA; Supplemental Table S17: VDD LF vs. VDD HF male, IPA; Supplemental Table S18: CTRL LF vs. CTRL HF female, IPA; Supplemental Table S19: VDD LF vs. VDD HF female, IPA.
