# Supplementary material for: Maternal Vitamin D Deficiency in Mice Increases White Adipose Tissue Inflammation in Offspring
Source: Cells. 2022 Jun 25;11(13):2024. doi: 10.3390/cells11132024 (PMC9265671; doi:10.3390/cells11132024)
Supplement: Supplementary file 1 [file cells-11-02024-s001.zip › cells-1713608-supplementary.pdf]

Supplemental Table S1. CRTL LF vs VDD LF male, IPA.

Ingenuity Canonical Pathways	p value	z-score	number of genes
LXR/RXR Activation	1,38E-05	0,707	9
HIF1α Signaling	0,0030903	0,707	8
LPS/IL-1 Mediated Inhibition of RXR Function	0,0281838	-1	7
AMPK Signaling	0,0075858	-0,447	8
cAMP-mediated signaling	0,0177828	-0,378	7
White Adipose Tissue Browning Pathway	0,0052481	0,816	6

Supplemental Table S2. CTRL HF vs VDD HF male, IPA.

Ingenuity Canonical Pathways	p value	z-score	number of genes
IL-8 Signaling	0,0371535	-0,277	15
LPS/IL-1 Mediated Inhibition of RXR Function	0,042658	0	17
HIF1α Signaling	1,995E-08	0,186	46
ErbB4 Signaling	0,0081283	0,816	8
ILK Signaling	9,772E-08	1,043	27
IL-6 Signaling	0,0467735	1,134	10
ERK/MAPK Signaling	0,0001288	1,213	22
PI3K Signaling in B Lymphocytes	0,0081283	1,265	13
NF-κB Activation by Viruses	0,047863	1,342	7
p38 MAPK Signaling	0,0295121	1,414	10
PPARα/RXRα Activation	0,0002291	1,604	20
ERK5 Signaling	0,0112202	1,633	8
IL-3 Signaling	0,0063096	1,89	9
Integrin Signaling	0,0003236	2,065	21
White Adipose Tissue Browning Pathway	1,95E-05	2,357	18

Supplemental Table S3. CTRL HF vs VDD HF female, GO.

GO biological process complete	Mus musculus - REFLIST (21988)	upload_1 (1935)	upload_1 (raw P-value)	upload_1 (FDR)
positive regulation of MAPK cascade	516	51	7,28E-04	2,43E-02
regulation of MAPK cascade	716	66	5,86E-04	2,06E-02

Supplemental Table S4. CTRL LF vs VDD LF female, IPA.

Ingenuity Canonical Pathways	p value	z-score	number of genes
LPS/IL-1 Mediated Inhibition of RXR Function	0,0070795	-1,414	21
LXR/RXR Activation	0,0046774	2,309	13
NRF2-mediated Oxidative Stress Response	0,0144544	2	19
Apelin Adipocyte Signaling Pathway	0,018197	1	9
VDR/RXR Activation	0,0031623	0,816	10

Supplemental Table S5. CTRL HF vs VDD HF female, IPA.

Ingenuity Canonical Pathways	p value	z-score	number of genes
LPS/IL-1 Mediated Inhibition of RXR Function	1,023E-09	-1,528	55
NRF2-mediated Oxidative Stress Response	0,0177828	2,714	32
IL-15 Production	0,0323594	-2,828	18
LXR/RXR Activation	0,0018197	0,535	22
p53 Signaling	0,0077625	0	17
White Adipose Tissue Browning Pathway	1,38E-05	-0,756	29
VDR/RXR Activation	0,0017378	0	16
Apelin Adipocyte Signaling Pathway	0,0019055	1,5	17

Supplemental Table S6. CTRL LF vs CTRL HF male, GO.

GO biological process complete	Mus musculus - REFLIST (21988)	upload_1 (1260)	upload_1 (raw P-value)	upload_1 (FDR)
response to cytokine	757	67	9,48E-04	3,76E-02
brown fat cell differentiation	39	12	1,35E-05	1,09E-03

Supplemental Table S7. VDD LF vs VDD HF male, GO.

GO biological process complete	Mus musculus - REFLIST (21988)	upload_1 (1260)	upload_1 (raw P-value)	upload_1 (FDR)
regulation of cytokine production involved in inflammatory response	51	13	2,13E-03	4,95E-02
negative regulation of response to cytokine stimulus	57	14	1,97E-03	4,66E-02
positive regulation of tumor necrosis factor superfamily cytokine production	102	25	5,92E-05	2,71E-03
positive regulation of tumor necrosis factor production	101	24	1,12E-04	4,54E-03
regulation of tumor necrosis factor superfamily cytokine production	169	37	1,00E-05	5,88E-04
regulation of tumor necrosis factor production	167	36	1,62E-05	8,81E-04
regulation of cytokine-mediated signaling pathway	112	24	4,49E-04	1,46E-02
regulation of response to cytokine stimulus	122	26	2,58E-04	9,17E-03
inflammatory response	464	87	7,72E-09	1,03E-06
cytokine-mediated signaling pathway	278	48	1,10E-04	4,48E-03
positive regulation of cytokine production	448	77	1,29E-06	9,73E-05
regulation of cytokine production	714	121	2,16E-09	3,34E-07
negative regulation of cytokine production	268	45	4,06E-04	1,35E-02
response to cytokine	757	120	6,95E-08	7,45E-06

cellular response to cytokine stimulus	658	101	2,64E-06	1,85E-04
regulation of ERK1 and ERK2 cascade	313	47	2,04E-03	4,79E-02
positive regulation of MAP kinase activity	209	37	4,41E-04	1,45E-02
regulation of MAP kinase activity	285	50	5,71E-05	2,64E-03
positive regulation of MAPK cascade	518	74	5,02E-04	1,60E-02
regulation of MAPK cascade	704	100	5,38E-05	2,51E-03
response to leptin	20	8	1,58E-03	3,98E-02
brown fat cell differentiation	39	14	8,11E-05	3,53E-03

Supplemental Table S8. CTRL HF vs CTRL LF male, IPA.

Ingenuity Canonical Pathways	p value	z-score	number of genes
IL-15 Production	0,0019498	3	9
Gαi Signaling	0,0041687	-0,447	9
Integrin Signaling	0,0093325	3	11
ILK Signaling	0,0147911	2,121	10
HGF Signaling	0,0301995	2	7
White Adipose Tissue Browning Pathway	0,0346737	2,646	7

Supplemental Table S9. VDD LF and VDD HF male, IPA.

Ingenuity Canonical Pathways	p value	z-score	number of genes
IL-8 Signaling	3,09E-05	4,796	25
CXCR4 Signaling	0,0001622	2,673	20
IL-15 Production	0,0008511	3,873	15
IL-7 Signaling Pathway	0,0012303	2,53	11
Inflammasome pathway	0,002138	2,236	5
p53 Signaling	0,0025704	0,302	12
Apelin Adipocyte Signaling Pathway	0,0083176	0,333	10
IL-2 Signaling	0,0085114	2,121	8
IL-9 Signaling	0,0251189	2	5
CCR3 Signaling in Eosinophils	0,0295121	2	12
HIF1α Signaling	0,0436516	3,5	16
Th2 Pathway	0,0001047	1,732	18
Fcγ Receptor-mediated Phagocytosis in Macrophages and Monocytes	0,0001585	3,742	14
PI3K/AKT Signaling	0,000631	2,646	21
Th1 Pathway	0,0022387	2,714	14
STAT3 Pathway	0,0056234	1,265	14
FcγRIIB Signaling in B Lymphocytes	0,0075858	2,646	10
ERK/MAPK Signaling	0,0077625	2,309	18
PI3K Signaling in B Lymphocytes	0,0091201	3,464	14
Gαs Signaling	0,0138038	2,828	12
Gαi Signaling	0,0158489	1,667	13
Gαq Signaling	0,017378	3,317	15
Noradrenaline and Adrenaline Degradation	0,0251189	2,236	5
CREB Signaling in Neurons	0,0251189	5,667	41
Gα12/13 Signaling	0,0269153	3,464	12

Integrin Signaling	3,802E-08	5,292	31
ILK Signaling	1,072E-06	3,4	27
Neuroinflammation Signaling Pathway	0,0014125	4,796	30

Supplemental Table S10. CTRL LF vs CTRL HF female, GO.

GO biological process complete	Mus musculus - REFLIST (21988)	upload_1 (1935)	upload_1 (raw P-value)	upload_1 (FDR)
regulation of interleukin-2 production	59	15	7,45E-04	2,24E-02
positive regulation of cytokine production	448	68	7,83E-05	3,36E-03
regulation of ERK1 and ERK2 cascade	313	47	1,20E-03	3,31E-02
response to cytokine	757	106	1,40E-05	7,82E-04
cellular response to cytokine stimulus	658	90	1,66E-04	6,35E-03
regulation of cytokine production	714	96	1,74E-04	6,58E-03
positive regulation of MAP kinase activity	209	39	5,53E-05	2,53E-03
positive regulation of MAPK cascade	518	87	1,38E-07	1,43E-05
regulation of MAP kinase activity	285	47	1,56E-04	6,02E-03
regulation of MAPK cascade	704	105	1,41E-06	1,05E-04

Supplemental Table S11. CTRL LF and CTRL HF female, IPA.

Ingenuity Canonical Pathways	p value	z-score	number of genes
Gαi Signaling	0,0066069	1,265	14
α-Adrenergic Signaling	0,030903	2,236	10
CCR3 Signaling in Eosinophils	0,0288403	2,646	12
Th1 Pathway	0,0144544	2,714	12
Chemokine Signaling	0,0380189	2,828	8
ERK/MAPK Signaling	0,0288403	2,887	17
White Adipose Tissue Browning Pathway	0,030903	2,887	12
Neuroinflammation Signaling Pathway	0,0301995	2,982	23
HIF1α Signaling	0,0229087	3,5	17
IL-15 Production	0,0008128	3,873	15
IL-8 Signaling	0,0029512	4,123	20

Supplemental Table S12. VDD LF and VDD HF female, IPA.

Ingenuity Canonical Pathways	p value	z-score	number of genes
Apelin Liver Signaling Pathway	2,239E-05	2	4
Hepatic Fibrosis Signaling Pathway	0,0004169	2,646	10

Supplemental Table S13. miRNA differentially regulated.

miR CTRL LF VDD LF males

mature ID	pval	Fold Change
mmu-miR-142a-5p	0,033762	0,7445
mmu-let-7d-5p	0,008579	1,685
mmu-miR-141-3p	0,031802	0,0419
mmu-let-7f-5p	0,009719	1,7523
mmu-miR-29a-3p	0,035893	1,4357

miR VDD LF VDD HF males

mature ID	pval	Fold Change
mmu-miR-29b-3p	0,005735	1,8142
mmu-miR-30e-5p	0,045846	1,4028
mmu-miR-30a-5p	0,04772	1,398
mmu-miR-29a-3p	0,008446	1,9846
mmu-miR-101a-3p	0,001021	2,8915

miR CTRL LF CTRL HF females

mature ID	pval	Fold Change
mmu-miR-30d-5p	0,01473	0,5886

miR CTRL LF VDD LF females

mature ID	pval	Fold Change
mmu-miR-29b-3p	0,032927	1,6249
mmu-miR-30e-5p	0,039019	1,6521
mmu-miR-322-5p	0,044697	2,2197
mmu-miR-146a-5p	0,019032	2,3236

miR VDD LF VDD HF females

mature ID	pval	Fold Change
mmu-miR-29b-3p	0,002694	3,9986

miR CTRL HF VDD HF females

mature ID	pval	Fold Change
mmu-miR-29a-3p	0,037866	0,5728

Supplemental Table S14. CTRL LF vs VDD LF male, IPA.

Ingenuity Canonical Pathways	PVALUE	number of genes
IL-6 Signaling	1,95E-05	7
IL-10 Signaling	0,0112202	3
IL-7 Signaling Pathway	0,0141254	3
ILK Signaling	0,040738	4
HIF1α Signaling	0,0457088	4

Supplemental Table S15. CTRL LF vs VDD LF female, IPA.

Ingenuity Canonical Pathways	PVALUE	number of genes
ILK Signaling	0,002884	8
IL-4 Signaling	0,0051286	5
STAT3 Pathway	0,0057544	6
ERK5 Signaling	0,0120226	4
IL-8 Signaling	0,0134896	7
IL-7 Signaling Pathway	0,0141254	4
p38 MAPK Signaling	0,0147911	5
JAK/STAT Signaling	0,0169824	4
Th1 and Th2 Activation Pathway	0,017378	6
Role of IL-17F in Allergic Inflammatory Airway Diseases	0,0186209	3
IGF-1 Signaling	0,0363078	4
IL-2 Signaling	0,0363078	3
HIF1α Signaling	0,0389045	6
IL-17A Signaling in Airway Cells	0,0457088	3
TGF-β Signaling	1,995E-05	8
ERBB4 Signaling	0,047863	3

Supplemental Table S16. CTRL HF vs VDD HF female, IPA.

Ingenuity Canonical Pathways	PVALUE	number of genes
IL-6 Signaling	6,026E-05	10
STAT3 Pathway	0,0004571	9
p38 MAPK Signaling	0,0009333	8
Role of MAPK Signaling in the Pathogenesis of Influenza	0,0025119	6
ERK/MAPK Signaling	0,0036308	10
ILK Signaling	0,0067608	9
IGF-1 Signaling	0,0081283	6
IL-8 Signaling	0,0091201	9
Chemokine Signaling	0,0109648	5
TNFR1 Signaling	0,0112202	4
IL-2 Signaling	0,0190546	4
CCR3 Signaling in Eosinophils	0,0263027	6

IL-10 Signaling	0,0323594	4
ERK5 Signaling	0,0354813	4
IL-17 Signaling	0,0380189	7
NF-κB Activation by Viruses	0,0416869	4

Supplemental Table S17. VDD LF vs VDD HF male, IPA.

Ingenuity Canonical Pathways	PVALUE	number of genes
TGF-β Signaling	8,913E-06	13
p38 MAPK Signaling	0,001349	11
Th1 and Th2 Activation Pathway	0,0029512	13
ERK/MAPK Signaling	0,0033113	15
STAT3 Pathway	0,0097724	10
Role of IL-17F in Allergic Inflammatory Airway Diseases	0,0151356	5
ILK Signaling	0,0234423	12
CCR3 Signaling in Eosinophils	0,025704	9
Chemokine Signaling	0,0389045	6

Supplemental Table S18. CTRL LF vs CTRL HF female, IPA.

Ingenuity Canonical Pathways	PVALUE	number of genes
IGF-1 Signaling	0,0002884	8
Integrin Signaling	0,0007244	11
ERK5 Signaling	0,0012589	6
Role of JAK2 in Hormone-like Cytokine Signaling	0,0020893	4
ERK/MAPK Signaling	0,0028184	10
IL-4 Signaling	0,0039811	6
TGF-β Signaling	0,0046774	6
Role of JAK1 and JAK3 in γc Cytokine Signaling	0,0051286	5
IL-23 Signaling Pathway	0,0064565	5
Role of JAK family kinases in IL-6-type Cytokine Signaling	0,0074131	3
IL-3 Signaling	0,0091201	5
Chemokine Signaling	0,0095499	5
JAK/STAT Signaling	0,0107152	5
ILK Signaling	0,0162181	8
IL-6 Signaling	0,0177828	6
CCR3 Signaling in Eosinophils	0,0223872	6
STAT3 Pathway	0,0223872	6
Role of MAPK Signaling in Promoting the Pathogenesis of Influenza	0,0363078	5
IL-7 Signaling Pathway	0,0371535	4

Supplemental Table S19. VDD LF vs VDD HF female, IPA.

Ingenuity Canonical Pathways	PVALUE	number of genes
Th1 and Th2 Activation Pathway	0,0001698	16
TGF-β Signaling	2,754E-06	14
STAT3 Pathway	0,0015849	12
p38 MAPK Signaling	0,042658	8
IL-8 Signaling	0,0436516	12
IL-3 Signaling	0,0446684	6
IL-23 Signaling Pathway	0,0165959	5
IL-17 Signaling	0,0199526	12
IL-15 Production	0,0070795	10
ERK/MAPK Signaling	0,011749	14
CCR3 Signaling in Eosinophils	0,0331131	9
